# Commentary on Shimoyama et al. (2012): three ontologies to define phenotype measurement data

**DOI:** 10.3389/fgene.2014.00093

**Published:** 2014-04-24

**Authors:** John M. Hancock

**Affiliations:** Department of Physiology, Development and Neuroscience, University of CambridgeCambridge, UK

**Keywords:** ontologies, phenotype, data representation, phenotype measurement, measurement ontologies

As human genomics moves into a mass-scale era, whereby millions of genome sequences will soon become available, new opportunities are opening up to use these very large samples better to understand the relationship between genotype and phenotype.

A fundamental problem for such studies in the past has been lack of standardization in the description of the phenotypes used. Not only have disease concepts often been confused with phenotypes (most diseases manifest numerous, distinct phenotypes which not only makes up the disease description but often can be observed in a number of diseases), but the concepts used have at times been vague (Hancock et al., 2009; Schofield et al., 2010).

In addition, model organisms are frequently used to study disease-, and more broadly phenotype-related phenomena in systems with applicability to humans but which are not subject to equivalent ethical problems or issues of data protection. A key requirement for future computational analysis of the relationship between genotype and phenotype in human will therefore be to include knowledge from model organisms (Hancock et al., 2009).

Significant progress has been made in using ontologies to describe human and model organism phenotypes in recent years. Many computational biology communities, serving particular model organism experimental communities, have developed approaches to the ontological description of phenotypes, often associated with community databases. As an example, the Mammalian Phenotype ontology (MP) (Smith and Eppig, 2012) was developed in association with the Mouse Genome Database (Blake et al., 2014) to facilitate consistent annotation of phenotypes associated with genomic differences. The MP, although originally developed for mouse, was subsequently applied to rat in the context of the Rat Genome Database (Nigam et al., 2013). For humans the Human Phenotype Ontology (HPO) (Kohler et al., 2014) has been developed to reflect the atomic features of diseases, initially making use of the OMIM (Online Mendelian Inheritance in Man) resource (Amberger et al., 2011).

A drawback of these approaches to ontological description, which make use of so-called pre-composed ontologies which are prepared in advance to the annotation process, is that they are unable to represent the full range of phenotypic observations including “normal” states, or of representing subtle differences or numerical values. To address this, an alternative approach, often known as the PATO (Phenotype and Trait Ontology) approach, has been developed (Bard and Rhee, 2004; Gkoutos et al., 2004, 2009). This aims to make use of a group of compatible ontologies to produce combinatorial expressions of the type:

*Entity **E** has attribute **A** of value **V** when measured in organism **O** using test **T** under conditions **C***.

where elements in bold are terms from an appropriate ontology. The full implementation of such an approach is yet to be realized, although starts have been made in databases such as Zfin (Howe et al., 2013) and Europhenome (Morgan et al., 2010).

A key missing element in such a compositional approach is standard descriptions of experimental methods and conditions. Over the last decade or so a number of ontological approaches to defining experimental conditions have been developed. The MGED Ontology (Whetzel et al., 2006b) was developed to underpin the fulfillment of the MIAME (Minimum Information About a Microarray Experiment) metadata criteria (Brazma et al., 2001). The HUPO (Human Proteome Organization) PSI (Proteomics Standards Initiative) Mass Spectroscopy Vocabularies (Mayer et al., 2013) facilitate the description of experiments in proteomics and mass spectroscopy. The Metabolomics Standards Initiative has established COSMOS (COordination of Standards in MetabOlomicS) (Steinbeck et al., 2012) to describe metabolomics experiments, making use of the ISA (Investigation/Study/Assay) framework (Sansone et al., 2012). FuGO (the Functional Genomics Investigation Ontology) was developed to provide a broader structure for functional genomics experiments (Whetzel et al., 2006a). EXPO (Soldatova and King, 2006) attempts to provide a higher level ontology to which such domain-specific ontologies can be integrated.

An important addition to the armory of ontological frameworks that can be used to describe phenotypes is provided by Shimoyama et al. (2012) who describe a set of three ontologies that can be used to describe clinical measurements, measurement methods and experimental conditions for traits common to rat and man (and, by extension, to other mammalian model systems such as mouse and, potentially, more distantly related species). Their approach extends the availability of experimental description ontologies in a whole new direction and, crucially the types of measurement they can describe using these ontologies are similar to those used in large-scale phenotyping experiments on mouse models of human disease (Hancock and Dobbie, 2014). Their ontology system can be used to describe both human and model mammal phenotyping measurements (**T** and **C** in the above expression). It therefore provides an underpinning component for the computational study of genotype-phenotype relations in humans and model mammals. At the same time it provides a valuable set of terms and relations to facilitate more systematic annotation and searching of phenotype terms across human and model organism databases. This opens up exciting new opportunities for the unified analysis of human and mouse disease and phenotype data.

## Conflict of interest statement

The author declares that the research was conducted in the absence of any commercial or financial relationships that could be construed as a potential conflict of interest.
